# Developmentally-Inspired Biomimetic Culture Models to Produce Functional Islet-Like Cells From Pluripotent Precursors

**DOI:** 10.3389/fbioe.2020.583970

**Published:** 2020-10-07

**Authors:** Raymond Tran, Christopher Moraes, Corinne A. Hoesli

**Affiliations:** ^1^Department of Chemical Engineering, McGill University, Montreal, QC, Canada; ^2^Department of Biomedical Engineering, McGill University, Montreal, QC, Canada

**Keywords:** pancreas, stem cells, mechanobiology, development, differentiation, microenvironment, islets

## Abstract

Insulin-producing beta cells sourced from pluripotent stem cells hold great potential as a virtually unlimited cell source to treat diabetes. Directed pancreatic differentiation protocols aim to mimic various stimuli present during embryonic development through sequential changes of *in vitro* culture conditions. This is commonly accomplished by the timed addition of soluble signaling factors, in conjunction with cell-handling steps such as the formation of 3D cell aggregates. Interestingly, when stem cells at the pancreatic progenitor stage are transplanted, they form functional insulin-producing cells, suggesting that *in vivo* microenvironmental cues promote beta cell specification. Among these cues, biophysical stimuli have only recently emerged in the context of optimizing pancreatic differentiation protocols. This review focuses on studies of cell–microenvironment interactions and their impact on differentiating pancreatic cells when considering cell signaling, cell–cell and cell–ECM interactions. We highlight the development of *in vitro* cell culture models that allow systematic studies of pancreatic cell mechanobiology in response to extracellular matrix proteins, biomechanical effects, soluble factor modulation of biomechanics, substrate stiffness, fluid flow and topography. Finally, we explore how these new mechanical insights could lead to novel pancreatic differentiation protocols that improve efficiency, maturity, and throughput.

## Introduction

Cell therapies involve the transplantation of human tissues or cells to treat illnesses that progressively damage or degrade functional tissues. These treatments are typically limited by access to a reliable and cost-effective cell source. For a successful transplant, allogeneic matching requires identification, procurement and transport of donor material; and variations in quality of the donated tissue may affect therapeutic potential. Therefore, the large-scale production of biological material for cell therapies is a rapidly growing area of interest. The primary goal of such research is to produce adequate quantities of functional therapeutic cells in a cost-effective manner.

Treatment of type 1 diabetes by islet transplantation is one example of a currently approved cell therapy. Here, donor islets are used to replace the insulin-secreting functionality lost from the autoimmune destruction of pancreatic beta cells. The Clinical Islet Transplantation Consortium trial (CIT-07) reported that 87.5% of patients were free from severe hypoglycemic events and achieved normal or near normal glycemic control at the 1 year end point (Foster et al., 2018). As with other cell therapies, the supply of donor islets greatly limits availability of this treatment. Islet transplantation typically requires a dose of greater than 5000 islet equivalents (IE) per kg body weight (Shapiro et al., 2017). To achieve the 500,000–1,000,000 IE/patient, islets are typically pooled from multiple deceased donors. Furthermore, the viability and function of the recovered islets is dependent on extraction techniques and post-mortem handling (Rosenberg et al., 1999; Paraskevas et al., 2000; Negi et al., 2012).

The production of insulin-secreting beta-like cells from pluripotent stem cells (PSCs) is a potential solution to the donor supply issues of islet transplantation. The first report of stem-cell derived insulin-producing cells via spontaneous differentiation was almost two decades ago and reported only 1–2% insulin^+^ cells (Assady et al., 2001). However, subsequent studies demonstrated that insulin staining and glucose-stimulated release can be due to insulin uptake from cell culture media (Rajagopal et al., 2003; Hansson et al., 2004; Sipione et al., 2004). Therefore, later studies reported C-peptide and/or pro-insulin content as well as more careful assessment of function and insulin granules instead of just insulin as a marker (Sipione et al., 2004; D’Amour et al., 2006; Naujok et al., 2009). More recent developments have been more successful, and rely on directed differentiation protocols which involve guiding stem cells through stages of pancreas development by mimicking the soluble signals present *in vivo*. These protocols are long and typically last more than 20 days (Rezania et al., 2014; Millman et al., 2016; Nair et al., 2019; Hogrebe et al., 2020). One of the first major breakthroughs was homogenous induction of PSCs into the endoderm lineage (up to 80% endoderm cells) (D’Amour et al., 2005) and subsequently into cell populations with significant C-peptide and proinsulin content (D’Amour et al., 2006). Since then, the efficiency of monohormonal beta cell induction has increased (∼40% NKX6.1^+^/C-peptide^+^) (Hogrebe et al., 2020). However, producing functional cell populations from PSCs remains an issue (Pagliuca et al., 2014; Rezania et al., 2014; Velazco-Cruz et al., 2019, 2020; Veres et al., 2019). Maturation of PSC-derived pancreatic progenitors can be accomplished when transplanted *in vivo* (Rezania et al., 2012; Robert et al., 2018) but typically requires cell aggregation *in vitro* (Toyoda et al., 2015; Nair et al., 2019). Hence, current strategies to improve directed differentiation protocols involve optimizing the duration of each differentiation stage as well as incorporating various aspects of the developmental microenvironment (Nostro et al., 2015; Mamidi et al., 2018; Nair et al., 2019; Hogrebe et al., 2020).

This review will primarily focus on recent biomimetic approaches which exploit biochemical and biomechanical cues to promote the differentiation of pancreatic cells. We will first address directed differentiation protocols relying on soluble factors, followed by a discussion of more recent advances which mimic biophysical features of the developmental microenvironment, by manipulating cell–cell or cell–substrate interactions.

## Directed Pancreatic Differentiation and Cell Signaling

**FIGURE 1:**
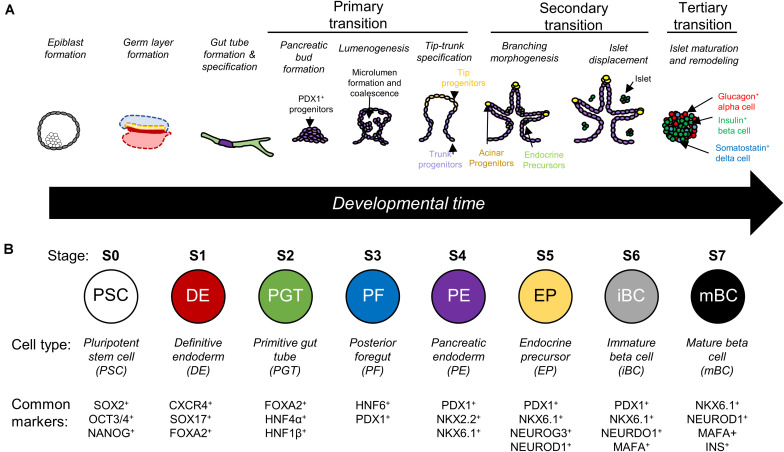
**(A)** Illustrative schematic of pancreas development which is characterized by three main transitions. Primary transition involves bud formation and specification of different pancreatic cell types. Secondary transition involves branching of the pancreatic bud, further specification of endocrine precursors, and the delamination of islet cells. Tertiary transition involves remodeling of islet architecture and further maturation (Pan and Wright, 2011; Benitez et al., 2012; Jennings et al., 2015; Dassaye et al., 2016). **(B)** Directed differentiation protocols recreate stages of differentiation in a step-by-step manner and look for expression of key transcription factors. Based on data presented by Rezania et al. (2014) and adapted from Tran et al. (2019).

Directed differentiation is the process of guiding stem cells through development to produce a desired, mature cell population. Classically, this is done by the timed addition of soluble factors to mimic conditions present during stages of development. In the context of pancreatic beta cell manufacturing, protocols emulate the multistep transition from pluripotent stem cells to definitive endoderm lineage, then toward the specification of the primitive gut tube and the subsequent pancreatic developmental steps (Figure 1A) (Pan and Wright, 2011; Benitez et al., 2012; Jennings et al., 2015; Dassaye et al., 2016). Each stage of development is accompanied by the nuclear expression of key transcription factors such as PDX1 or NKX6.1, which are commonly accepted as the first pancreatic and beta cell lineage markers respectively (Figure 1B) (Offield et al., 1996, 1; Schaffer et al., 2013, 1). Ultimately, the end goal of these protocols is to produce monohormonal, insulin-producing cells that have glucose-sensing capability comparable to native islets.

In addition to soluble biochemical signals, other components of the cellular microenvironment are known to play a critical role during embryonic development. Stimuli from the microenvironment include biophysical cell–cell interactions and cell–extracellular matrix (ECM) interactions (Discher et al., 2009), which can interact with soluble factor signaling in a synergistic manner. However, the *in vivo* microenvironment, particularly during embryonic development, is particularly complex and difficult to mimic with current knowledge and culture systems.

Embryonic development is guided via highly dynamic signals from the surrounding cell microenvironment with remarkable precision and robustness. As cells differentiate, they relay different signals to neighboring cells by secreting soluble factors and matrix proteins. The soluble signaling cues associated with pancreatic differentiation have been well-studied using animal models and include the Wnt, Activin/Nodal, fibroblast growth factor (FGF), bone morphogenetic protein (BMP), retinoic acid, and sonic hedgehog (Shh), and Notch signaling pathways (Hashemitabar and Heidari, 2019). However, relatively little attention has been paid to the physical stimuli present during embryonic development. Biomechanics and cell/tissue mechanobiology play a large role in guiding cell behavior especially during early embryogenesis (Heisenberg and Bellaïche, 2013). The pathways through which biomechanical cues translate to differentiation are not as well-understood *in vivo*. Studies of developmental mechanobiology are challenging, and these parameters are often not considered as it is unclear what mechanics are present during human embryogenesis. Furthermore, the complexity of the *in vivo* microenvironment makes it difficult to control these biomechanical signals and to delineate their effects on differentiation from other correlated stimuli. Therefore, the field relies mainly on studies of biochemical pathways with *in vivo* mouse models or *in vitro* human models for information.

**FIGURE 2:**
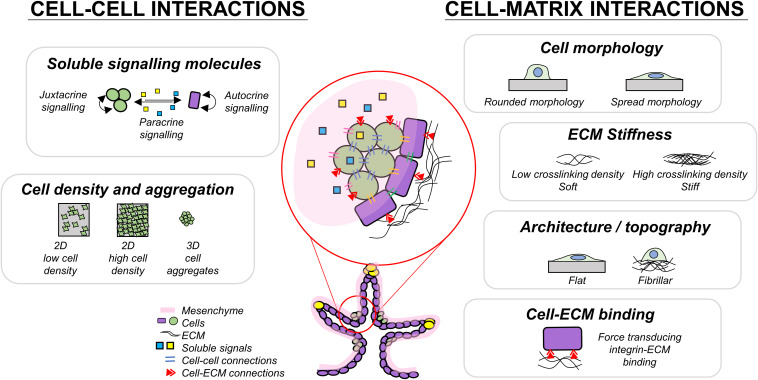
Summary of some cell interactions present during pancreas development which may play a role in guiding endocrine differentiation.

Cells respond to mechanical stimuli through mechanotransduction mechanisms, in which biomechanical stimuli are converted into biochemical signals (Chen, 2008; Moraes et al., 2011; Martino et al., 2018; Wolfenson et al., 2019). Reciprocally, cells alter the mechanics of their surrounding tissues by exerting contractile forces (Wozniak and Chen, 2009; Wang H. et al., 2014) and depositing or degrading the ECM proteins (Rozario and DeSimone, 2010; Bonnans et al., 2014). External biomechanical stimuli can promote cytoskeletal reorganization and subsequent changes in protein activity or localization, gene expression (Wang et al., 2009), proliferation (Frank et al., 2016), and differentiation (Saha et al., 2006; Fang et al., 2019). Mechanotransduction could occur via mechanosensitive pathways such as Hippo signaling (Piccolo et al., 2014) which is heavily involved in development by regulating tissue growth (George et al., 2012), death (Sharma et al., 2017), and cell fate (Rosado-Olivieri et al., 2019). Properly designed novel cell culture substrates to control these aspects of cell–cell and cell–environment interactions could help drive cells toward desired cell fates. Biomechanical cues could be incorporated into cell culture systems to reduce the cost of soluble factors added in differentiation or the time required to produce a desired cell type. Therefore, in the following sections, we review the interactions between cells and biophysical components in the developmental microenvironment, to build a roadmap towards more advanced beta stem cell specification platforms (Figure 2).

## Cell–Cell Interactions

Resident cells communicate with their neighbors during development by secreting soluble signaling molecules which can bias differentiation (Basson, 2012). The developmental microenvironment contains multiple transient cell types which each have different roles in the differentiation process. Within this environment, cells actively uptake soluble signaling factors and nutrients to form concentration gradients which are dependent on tissue thickness, vascularization, and oxygenation. In each of the following sections, we describe approaches to pancreatic differentiation that rely on controlling the soluble factor environment, optimizing cell seeding density, and forming pancreatic aggregates, all of which mimic aspects of developmental processes.

### Soluble Factor-Based Directed Differentiation

The *in vivo* cellular microenvironment is much more intricate than can be modeled by current *in vitro* systems. Although directed differentiation protocols that focus solely on the timed addition of soluble signaling factors can generate insulin-producing cells (Pagliuca et al., 2014; Rezania et al., 2014; Millman et al., 2016; Yung et al., 2019; Hogrebe et al., 2020), these protocols typically produce some polyhormonal cells which have a transcriptome similar to fetal beta cells (Hrvatin et al., 2014), while monohormonal insulin-producing cells are more similar to adult pancreatic beta cells (Pagliuca et al., 2014). This may explain why earlier protocols produced cells with impaired glucose-sensing capabilities (Rezania et al., 2014), which may be due to metabolic bottlenecks in glycolysis (Davis et al., 2020). Furthermore, variation in differentiation efficiency between PSC lines (Osafune et al., 2008; Nostro et al., 2015) and in handling procedures between research groups represents a significant challenge for clinical translation. Interestingly, when immature NKX6.1^+^ pancreatic progenitors are transplanted into immunocompromised mice, they form functional insulin-producing cells (Kroon, 2008; Rezania et al., 2012) and are able to provide long-term glycemic control when encapsulated within immunoprotective alginate polymers (Vegas et al., 2016). This suggests that some missing elements of the *in vivo* microenvironment have the potential to further improve mature beta cell production for therapeutic use.

The presence of other cell types found in native islets can improve insulin response. Glucose-stimulated insulin secretion is regulated by paracrine glucagon signaling from neighboring alpha cells (Samols et al., 1965; Trimble et al., 1982; Kelly et al., 2011; Svendsen et al., 2018). Human islets that are dispersed and reaggregated into controlled-size aggregates can restore glycemic control in diabetic mice, suggesting that functional insulin response can be achieved with the appropriate co-culture (Yu et al., 2018). Soluble factors from mesenchymal stromal/stem cells (MSCs) in Transwell^®^ cultures also improves proliferation, pancreatic differentiation, and engraftment function via IGF1 signaling (Li et al., 2019). The presence of human amniotic epithelial cells within islet organoids grafted into a type 1 diabetes mouse model had enhanced blood glucose control and increased percentage diabetic reversal (96 vs. 16%) after 1 month compared to animals transplanted with only islet cells (Lebreton et al., 2019). In directed differentiation, the addition of endothelial cells has also been shown to induce maturation of hESC-derived pancreatic progenitor cells toward insulin-expressing cells (Jaramillo et al., 2015). Overall, the addition of other cell types may improve current differentiation protocols, but it is currently not well-understood whether these effects originate from paracrine signaling or cell–cell contacts.

Cell proliferation and other cell attributes such as metabolic rates can change the soluble factor microenvironment, including the level of dissolved oxygen available to cells. Control of oxygen and other nutrient levels therefore might be a useful strategy to improve cell functionality. Beta cells have a high oxygen demand and the oxygenation of islets is critical for survival (Komatsu et al., 2017) and function (Ludwig et al., 2012). Differentiation of PSCs under increased oxygen tension promoted differentiation into pancreatic progenitors and subsequently, insulin-producing cells over normoxic conditions (Heinis et al., 2010; Hakim et al., 2014). Bioreactors providing feed-back control over nutrient and other biomolecular concentrations could improve the reproducibility of PSC pancreatic differentiation protocols.

### Cell Density Effects on Differentiation and Function

Regulation of cell density is paramount to achieve controlled differentiation toward the desired cell lineage. High initial cell seeding densities promotes the differentiation of PSCs into definitive endoderm cells (Gage et al., 2013) as well as into downstream endocrine lineages (Takizawa-Shirasawa et al., 2013). Pancreatic differentiation arising from high cell density protocols is correlated with the downregulation of Rho-associated kinases (ROCK) and non-muscle myosin II (NM II) mRNA and proteins. This effect was independent of cell proliferation. At similar cell densities, inhibition of ROCK-NM II pathways improved pancreatic differentiation over controls, suggesting that this effect is due to aggregation (Toyoda et al., 2017). High cell density cultures may therefore better recapitulate the local cell density in the developing pancreas, thereby increasing bias toward pancreatic endocrine fate. The mechanism by which high density cultures promotes pancreatic differentiation may include increased cell-to-cell contact, changes in oxygen tension, or increased soluble factor signaling. While the exact mechanisms remain to be elucidated, this general approach may be valuable for pancreatic differentiation protocols.

### Cell Aggregation

#### Effects of Cell Aggregation on Beta Cell Differentiation and Function

Forcibly aggregating pancreatic cells could capture many of the effects of high density culture, and may also better mimic the 3D environment and cell–cell signaling found *in vivo* to ultimately produce a more relevant phenotype *in vitro* (Antoni et al., 2015; Hohwieler et al., 2017; Glieberman et al., 2019). *In vitro*, spontaneous, cell-mediated clustering of pancreatic endoderm cells has been observed in 2D cultures (Nostro et al., 2015; Tran et al., 2020). A potential candidate driving this clustering behavior *in vivo* is the secretion and chemotaxis of FGF2, which further promotes differentiation into hormone producing, islet-like clusters (Hardikar et al., 2003). Thus, most directed differentiation protocols to generate functional beta-like cells rely on culturing differentiating and maturing pancreatic endoderm cells as aggregates (Pagliuca et al., 2014; Nair et al., 2019). The benefits of forced aggregation in directed differentiation may have origins in the biomimicry of pancreatic development where cell clusters are formed in primary transition and islet clustering.

Cell aggregation is important for proper functionality of beta cells (insulin response to glucose challenges) perhaps due to improved paracrine signaling and cell–cell contacts (Chowdhury et al., 2013; Lecomte et al., 2016). Paracrine signaling is necessary for proper crosstalk between the various hormonal cells (alpha, beta, delta, gamma cells) in native islets (Caicedo, 2013). Insulin secretion is also aided by calcium-driven electrical coupling of islet cell clusters (Pedersen et al., 2005; Sabatini et al., 2019). PSC-derived pancreatic aggregates encapsulated within a 3D matrix showed increased insulin secretion after 6 days compared to encapsulated single cells and cells cultured on a flat 2D surface, suggesting these functionalities may be closely linked to aggregation and paracrine signaling (Kim et al., 2019). Forced aggregation of PSC-derived pancreatic progenitor cells increases the proportion of NKX6.1^+^ cells over standard 2D cultures (Toyoda et al., 2015). In their later work, Toyoda et al. (2017) demonstrated that inhibition of Rho-associated kinases (ROCK) and non-muscle myosin II (NM II) increased the fraction of NKX6.1^+^ cells by emulating an aggregate phenotype even in low density cultures, suggesting that the physical act of aggregation may not be necessary. In addition to this, recent studies have shown that insulin-secreting cells can be produced in planar, non-aggregate protocols by the timed depolymerization of the actin cytoskeleton (Hogrebe et al., 2020).

#### Technologies to Study Cell Aggregation

Proper control of aggregate size is an important design consideration since mass transfer limitations arise in larger aggregates and gradients in soluble signaling factors begin to play a role in specification. *In vitro*, larger non-vascularized islets may develop central necrosis due to high metabolic demand and low oxygenation (Avgoustiniatos and Colton, 1997; Komatsu et al., 2017). To avoid core hypoxia, finite element models can be used to determine the optimal aggregate size in suspension (Buchwald, 2009) and encapsulation systems (Avgoustiniatos and Colton, 1997; Song and Millman, 2016). The average diameter of human islets is around 100–400 μm (Hellman, 1959; Suszynski et al., 2014; Ionescu-Tirgoviste et al., 2015). When islets and stem cell-derived beta cells are dissociated and re-aggregated, the optimal diameter to maintain viability and function was shown to be 100–150 μm (Hilderink et al., 2015; Song and Millman, 2016) while also improving survivability and function, perhaps due to improved nutrient availability (Yu et al., 2018). Therefore, creating aggregates of uniform size is crucial for reproducible production of functional insulin-producing cells.

Several groups have developed methods to reproducibly create endocrine cell aggregates of defined size to improve functionality (Gao et al., 2019; Nair et al., 2019; Velazco-Cruz et al., 2019). Culturing pancreatic cells on microporous scaffolds can improve differentiation toward insulin secreting beta-like cells over suspension cultures by guiding formation of consistently sized aggregates (Youngblood et al., 2019). Physical confinement of 3D MIN6 aggregates in 2% alginate beads promotes glucose-stimulated insulin secretion but decreases proliferation compared to adherent 2D culture (Hoesli et al., 2011). Micropatterned culture can be used to promote cell-driven clustering of PSC-derived NKX6.1^+^ cells with defined sizes while maintaining ease of handling associated with 2D cultures. Clustered cells from micropatterned confinement have increased PDX1 and NKX6.1 protein expression when compared to unconfined cells and this effect was further correlated with local cell density increases (Tran et al., 2020). Agarose microwells (Hilderink et al., 2015), pyramid-shaped polystyrene wells (Ungrin and Zandstra, 2011), or hydrogel micropockets (Zhao et al., 2019) have also been used to generate cell aggregates of controlled size. The production of uniformly sized cell aggregates in a scalable matter may be the key to producing mature cells for cell therapies in a consistent manner. Within these aggregates, cells deposit insoluble ECM proteins and dynamically change the surrounding microenvironment, which suggests that the architecture of these clusters and in general, the surrounding surfaces could play a large role in differentiation.

## Cell–Matrix Interactions

The ECM presents cues to surrounding cells in the form of biochemical signals, growth factors, and biophysical signals (Nakayama et al., 2014). Native ECM found *in vivo* is extremely complex and contains 100s of different proteins with varying composition depending on region. Combinatorial protein arrays are useful to study the interactive and additive effects of different ECM components on stem cell behavior (Flaim et al., 2005; Alberti et al., 2008; Guilak et al., 2009; LaBarge et al., 2009). However, the architecture of native ECM is equally important and also plays a role in guiding stem cell differentiation through cell-generated force feedback mechanisms (Trappmann et al., 2012). In the developing embryo, the ECM structure and composition is dynamically evolving, which constantly changes the biochemical and biophysical cues presented to developing cells (Rozario and DeSimone, 2010). Understanding the different aspects of the ECM and their role in differentiation during each stage of pancreas development (Table 1) could help the development of novel biomimetic culture systems to better guide the production of functional insulin-secreting cells.

**TABLE 1:** Compiled effects of substrate stiffness and architecture on various pancreatic differentiation platforms.

Author	Cell type	Differentiation type	Platform	ECM coating	Apparent modulus (kPa)	Cell morphology	Relative stiffness	Impact
***Architecture***								
Ghanian et al., 2015	hESCs	Directed S0 → S1	Electrospun poly(ε-caprolactone)	Matrigel	Not reported	Clumped	–	hESCs cultured on small diameter nanofibers adopted a clumped morphology and had improved definitive endoderm differentiation.
			Tissue culture polystyrene (control)	Matrigel	3 × 10^6^*	Spread	Stiff	
Maldonado et al., 2017	iPSCs	Directed S0 → S3	Electrospun poly(ε-caprolactone)	Col I	20	Round, 3D colonies +	Soft	“Soft” nanofibers promoted posterior foregut and pancreatic differentiation. “Stiff” surfaces promoted mesodermal differentiation while downregulating pancreatic differentiation.
			Electrospun polyether-ketone-ketone	Col I	300	Spread, flattened 2D colonies +	Stiff	
			Tissue culture polystyrene (control)	Col I	3 × 10^6^*		Stiff	
***Substrate stiffness***								
Narayanan et al., 2013	hESCs	Spontaneous differentiation	Hyaluronic acid hydrogels	Col IV, Fn, Lam	1.3–3.5	Not reported	Soft	Optimal differentiation with 2.1 kPa gels and a 1/3/3 mixture of Col IV, fibronectin, and laminin.
			Tissue culture polystyrene (control)	RIN5F ECM	3 × 10^6^*	Not reported		
Rasmussen et al., 2016b	hESCs	Directed S0 → S3	High aspect ratio polycarbonate nanopillars	Fn	34.6	Small, tight 2D clusters. Elongated and aligned with nanopillars	Soft	Poor hESC adhesion on soft nanopillars. “Soft” surfaces promoted endoderm (S1) differentiation. Control had significantly higher PDX1 protein expression (S3) compared to test conditions.
			Low aspect ratio polycarbonate nanopillars	Fn	2800	Spread	Stiff	
			Tissue culture polystyrene (control)	Fn	3 × 10^6^*	Spread	Stiff	
Richardson et al., 2016	hESCs	Directed S0 → S3	Low concentration barium alginate capsules	N/A	3.9 ± 1.3	Larger circular 3D colonies	Soft	“Soft” capsules increase hESC proliferation and were highly PDX1^+^ (S3). Localized deposition of Col I and Lam in “soft” capsules. “Stiff” capsules support endodermal (S1) differentiation but downregulates pancreatic differentiation.
			High concentration barium alginate capsules	N/A	73.2 ± 22.4	Small, growth restricted 3D colonies and large, elongated 3D colonies	Stiff	
Kim et al., 2019	Rat islets and PSCs	Directed S0 → S7	dECM bio-ink	Human pdECM	3	Not reported	-	Insulin secretion and maturation were enhanced in cells cultured in pdECM bioinks compared to 2D tissue culture polystyrene (TCPS), alginate gels, and collagen gels
			Collagen gel	Col I	100	Not reported	–	
			Tissue culture polystyrene (control)	–	3 × 10^6^*		–	
Hogrebe et al., 2020	PSCs	Directed S0 → S7	Tall collagen I gel	Col I	N/A	Not reported	Soft	Decreased stiffness via increasing gel height promotes overall endocrine induction (increases *NGN3, NKX2.2, NEUROD1* and decreases *SOX9, NKX6.1* expression)
			Short collagen I gel	Col I	N/A	Not reported	Stiff	
Pennarossa et al., 2018	Mouse dermal fibroblasts	Transdifferentiation	Polyacrylamide hydrogels	Col I	0.1	2D epithelioid structure in small, scattered clusters	Soft	Improved transdifferentiation toward monohormonal pancreatic endocrine cells on soft substrates
			Tissue culture polystyrene (control)	Col I	3 × 10^6^*	3D spherical structures	Stiff	

Fn, fibronectin; Lam, laminin; Col, collagen; pdECM, pancreatic decellularized extracellular matrix. *Stiffness of polystyrene shown for reference taken from Gilbert et al. (2010). ^+^Cell morphology details taken from previous work from same group (Maldonado, 2015).

### Biochemical Effects of ECM Proteins

The presence of ECM proteins produced within a cell and ECM pre-adsorbed or conjugated onto culture substrates, is essential for successful differentiation. These proteins can also bind growth factors which can affect presentation to the cells and improve growth factor stability (Taipale and Keski-Oja, 1997; Belair et al., 2014). The ECM components of the microenvironment can influence survival, adhesion, proliferation, and guide downstream cell fate decisions (Guilak et al., 2009; Gattazzo et al., 2014). The ECM of the human adult islet is composed mainly of laminin, collagen IV, fibronectin, and other types of collagen; whereas the embryonic pancreas ECM is fundamentally distinct and is primarily comprised of vitronectin, fibronectin, and collagen IV (Stendahl et al., 2009). Designing cell culture systems with combinations of ECM proteins found in the pancreas could therefore help improve pancreatic cell function and differentiation (Table 1, Column 5).

Several works have shown that islets and other pancreatic cells have improved functionality when cultured in matrices that recapitulate those found *in vivo*. Collagen and fibronectin have been shown to promote beta cell survival, while laminins are responsible for beta cell specification and insulin secretion (Arous and Wehrle-Haller, 2017). Islets encapsulated in hydrogels containing collagen IV and laminin show improved glucose-stimulated insulin secretion response (Weber and Anseth, 2008; Kim et al., 2019). Incorporating ECM-derived peptides such as RGD, LRE, PDSGR has also been shown to improve viability and glucose-stimulated insulin secretion in alginate encapsulated islets (Llacua et al., 2016; Medina et al., 2020).

Coating cell culture substrates with ECM proteins to mimic the biochemical effects of the ECM can guide directed differentiation of PSCs toward the endocrine lineage. Directed differentiation of PSCs in 2D conditions usually begins on a Matrigel-coated surface (Rezania et al., 2014; Hogrebe et al., 2020; Tran et al., 2020). However, Matrigel suffers from batch-to-batch variability which could affect differentiation outcomes. Purified ECM proteins (Amit et al., 2004; Ludwig et al., 2006), recombinant proteins (Chen et al., 2011; Wang Y. et al., 2014), or synthetic matrices (such as functionalized polyethylene glycol (PEG) or alginate) (Aisenbrey and Murphy, 2020) could serve as alternatives to Matrigel for PSC maintenance. Synthetic matrices and recombinant protein alternatives require more study in the context of pancreatic differentiation. Therefore, understanding the effect of constituent proteins in Matrigel and their effect on pancreatic differentiation is a necessary step to effectively guide differentiation outcomes. The synergistic role of various ECM protein mixtures can be screened combinatorically using protein arrays thus allowing for optimization of pancreatic differentiation protocols. For example, PSCs cultured on substrates coated with collagen I or in combination with collagen II and fibronectin reduced the time required for high purity endoderm differentiation (Rasmussen et al., 2016a). Fibronectin has previously been shown to downregulate the pluripotency of PSCs and enhance endoderm differentiation, which leads to the pancreatic lineage downstream (Brafman et al., 2013; Taylor-Weiner et al., 2013). Combinations of the main pancreatic ECM components, including fibronectin, collagen IV, and laminin, further promote ESC differentiation into pancreatic lineages (Narayanan et al., 2013; Malta et al., 2016). Further downstream, the composition of ECM protein coatings can also guide pancreatic cell fate decisions. Fibronectin-coated surfaces promote differentiation to ductal lineage whereas adhesion onto laminin-coated surfaces guides endocrine differentiation into NGN3^+^ cells (Mamidi et al., 2018).

Although Matrigel alternatives can be used to promote the initial stages of differentiation, downstream cell types may require a more complex environment. Embryonic murine pancreatic progenitors cultured in Matrigel gels could form organoid structures but not on synthetic 3D matrix alternatives (Greggio et al., 2013). PEG-based hydrogels functionalized with laminin 1 facilitated maintenance and expansion of murine pancreatic progenitors but on non-functionalized synthetic hydrogels, murine pancreatic progenitors did not expand and lost pancreatic character (Greggio et al., 2013). Similarly, bioinert PEG hydrogels maintained the viability of rat-derived pancreatic progenitors for 7 days, but failed to obtain glucose-responsive behavior, further suggesting the importance of ECM interaction (Mason and Mahoney, 2008; Amer et al., 2015). Pancreatic progenitor cells isolated from adult murine pancreas can also be cultured in methylcellulose (Jin et al., 2016) or laminin gels (Jin et al., 2013) to generate insulin-positive cells.

Separating the principal components of the ECM may lead to more efficient and cost-effective differentiation but synergistic effects of the complete matrix may be lost. Differentiation could be improved by using decellularized pancreatic ECM (dpECM), thus retaining ECM proteins composition. Utilizing decellularized pancreas matrix scaffolds in bioartificial pancreas designs could also be a fruitful strategy to improve islet functionality by retaining pancreas stiffness and matrix architecture (Goh et al., 2013; Guruswamy Damodaran and Vermette, 2018). Hydrogels composed of decellularized human pancreata can support the proliferation and differentiation of pancreatic progenitors into insulin-positive cells (Wan et al., 2017; Sackett et al., 2018). Pancreatic tissues derived from iPSC cultured in 3D bioprinted inks primarily composed of dpECM had increased Pdx1, insulin, and glucagon expression compared to collagen controls (Kim et al., 2019). Cell culture with the addition of collagen V, which was present in dpECM but not in Matrigel, enhanced islet organoid generation and glucose-responsive function (Bi et al., 2020). Overall, designing cell culture systems which recapitulate the *in vivo* ECM protein landscape could have significant impact to produce functional PSC-derived beta cells.

In addition to the combinatorial effects of individual ECM components, the composition of the ECM is dynamically changing during development. While one composition may promote early pancreatic differentiation, the same composition may hinder downstream differentiation stages. During hESC differentiation, ECM and MMPs are secreted which remodels the exogenous ECM on the culture substrate (Brafman et al., 2013). PSCs cultured on substrates supplemented with ECM proteins did not significantly improve beta cell maturation over 1–2 weeks, perhaps due to the contribution of cell-deposited ECM proteins. In self-organized PSC-derived beta cell clusters, cells deposit ECM which is largely composed of collagen IV, laminin, and fibronectin (Youngblood et al., 2019). This suggests guiding cell differentiation with ECM may not be a fruitful strategy in 3D aggregates or over longer time scales but could be used to improve initial differentiation after seeding.

### Biomechanical Effects

#### Soluble Factor-Based Modulation of Mechanotransducive Machinery

More recent differentiation strategies can achieve dynamic glycemic control in PSC-derived cells has been achieved by aggregating insulin-positive clusters (Nair et al., 2019; Velazco-Cruz et al., 2019), maturation through transplantation (Motté et al., 2014; Robert et al., 2018), or by timed activation of TGF-β signaling in aggregates (Velazco-Cruz et al., 2019). All these promising protocols present a combination of biochemical and biomechanical stimuli applied to the PSC-derived pancreatic progenitor cells. Mechanotransduction occurs via complex machinery within a cell that involves interplay between ECM connections (e.g., integrins, adherens), cytoskeletal organization, and the nuclear envelope (Eyckmans et al., 2011). Rather than mimic soluble factors present *in vivo*, a recent successful strategy has been to target specific mechanotransduction-associated pathways during directed differentiation to promote pancreatic differentiation and thus improve the capacity to produce insulin-secreting cells. Such factors include H1152 (a ROCK II inhibitor) (Ghazizadeh et al., 2017), verteporfin (Rosado-Olivieri et al., 2019), and latrunculin A (disrupts microfilament organization) (Hogrebe et al., 2020). Nuclear activation of YAP, which affects the mechanosensitive Hippo pathway, is inversely correlated with expression of pancreatic and endocrine markers, PDX1 and NGN3 respectively (Mamidi et al., 2018). Timed inhibition of YAP with verteporfin enhances endocrine differentiation while depleting the pancreatic progenitor population which may have benefits for downstream transplantation (Rosado-Olivieri et al., 2019). Depolymerizing the actin cytoskeleton with latrunculin A allowed endocrine differentiation that was previously not possible in 2D adherent cultures, perhaps suggesting that mechanosensing via the cytoskeleton is important in this process (Hogrebe et al., 2020). Altogether, this suggests that components of the *in vivo* cellular microenvironment are crucial for differentiation protocols and investigating how cells interact the surrounding extracellular matrix may be important in improving pancreatic differentiation protocols.

#### Biomechanical Cell Interactions

Cells interact with ECM proteins by binding with integrin receptors spanning across the cell membrane. These receptors can then activate downstream integrin-related signaling pathways that alter cell function and bias cell specification. In pancreas development, integrin-ECM signaling regulates collective cell migration and function (Rosenberg et al., 1999; Hammar et al., 2004; Shih et al., 2016). When pluripotent, hESCs express integrin domains α6 and β1 for laminin binding but this is downregulated after definitive endoderm differentiation (Wong et al., 2010). Differentiation toward definitive endoderm lineage with fibronectin and vitronectin is regulated by increased expression of integrin receptors α5, αV, β5 (Wong et al., 2010; Brafman et al., 2013). Further downstream, ECM-integrin α5 signaling associated with fibronectin binding promoted differentiation to pancreatic duct while disruption of this pathway enhanced endocrine differentiation (Mamidi et al., 2018). Although not yet studied in the context of mechanosensing for pancreatic differentiation, other surface receptors, such as stretch-activated ion channels (Liu et al., 2015; Nourse and Pathak, 2017), growth factor receptors (Tschumperlin et al., 2004), and cadherins (Ganz et al., 2006; Mui et al., 2016) could also play a role in guiding differentiation.

The surrounding ECM matrix also propagates stress (Wang H. et al., 2014) which allows cells to sense biomechanical cues through ECM-bound integrins (D’Angelo et al., 2011). Cells can also apply traction forces on the ECM through their integrin connections. Cells with high spread area also generate higher tractions forces (Rape et al., 2011; Han et al., 2012). Thus, these biomechanical interactions with ECM proteins may guide pancreatic differentiation through cell-generated forces caused by changes in the actin cytoskeleton organization. Integrin signaling drives cell-generated traction forces which are required for endoderm specification (Taylor-Weiner et al., 2015). Pathway analysis of single hiPSC-derived pancreatic cells encapsulated in alginate further suggests that integrin-signaling is involved in the cells’ ability to transduce mechanical confinement toward islet differentiation pathways (Legøy et al., 2020).

Cell morphology is guided by the ECM proteins of the culture substrate (Watt, 1986; Polte et al., 2004) which could affect differentiation by regulating gene expression through changes in endogenous tension (McBeath et al., 2004; Kilian et al., 2010; Lee et al., 2016). In differentiation of NGN3^+^ endocrine progenitors, fibronectin promotes high cell spread area while laminin-coated surfaces were associated with lower spreading (Mamidi et al., 2018). Aside from these biochemical signaling cues, cell behavior is dependent on the fibrous architecture and the apparent stiffness of the ECM, which both provide biomechanical cues to influence cell behavior. Therefore, it is important to try to decouple the specific ECM protein effects from the structural or mechanical stimuli provided by the ECM.

#### Fluid Flow

Pancreatic islets are highly vascularized tissues which is important for efficient oxygenation and rapid insulin response (Jansson and Hellerström, 1983; Carlsson et al., 1998). Microfluidics can resolve time-dependent secretion and metabolism of islets which would normally not be possible with conventional static cultures (Rocheleau et al., 2004; Wang et al., 2010; Nourmohammadzadeh et al., 2016; Walker et al., 2020). When cultured directly under high external flow rates, shear stress damages peripheral islet cells, reducing glucose-stimulated metabolism and calcium response (Shenkman et al., 2009; Sankar et al., 2011; Silva et al., 2013). However, blood flow (Wang et al., 2010) and islet culture in bioreactors (Minteer et al., 2014) also improves mass transfer to pancreatic islets (Wu et al., 2014), improving islet survival and function (Lock et al., 2011; Sankar et al., 2011) – particularly if the islets are protected from shear stress (Jun et al., 2019). Islets with endothelial cells co-cultured under external flow have higher endothelial cell survival compared to static culture which may improve islet functionality and health *ex vivo*, perhaps by mediating regeneration of islet ECM within aggregates (Sankar et al., 2011; Jun et al., 2019). Culture with low flow rates permits paracrine signaling between neighboring islet hormone cells and the low shear rates may help microvilli maintenance, which was correlated to insulin secretion capabilities (Bendayan, 1992; Geron et al., 2015; Jun et al., 2019).

Signals from pancreatic vasculature are critical in specifying differentiation (Lammert et al., 2001) but the role of shear stress on differentiation is not well-understood. There are many reports of pancreatic differentiation within stirred bioreactors (Schulz et al., 2012; Pagliuca et al., 2014; Mihara et al., 2017; Yabe et al., 2019) however little has been done to characterize the effect of shear stress on pancreatic differentiation within these systems. The differentiation of embryoid bodies under external flow promoted expression of beta cell specific markers, such as *NKX6.1* and *INS*, and improved glucose-stimulated insulin secretion sensitivity over static conditions (Tao et al., 2019). In the future it would be interesting to further investigate the biophysical effects of varying fluid flow rates on pancreatic differentiation and functionality.

#### Substrate Stiffness

Cells interact with aspects of the ECM, such as stiffness, by anchoring and pulling on the substrate. These signals are then transmitted through intracellular structures, such as the actin cytoskeleton, leading to downstream effects which alter cell fate decisions. Engler et al. (2006) showed that the differentiation of mesenchymal stem cells toward neurogenic, myogenic, and osteogenic lineages could be controlled by culturing cells on substrates of physiologically relevant *in vivo* stiffness. High stiffness environments reduce insulin expression of MIN6 cells confined in 3D polyacrylamide scaffolds (Nyitray et al., 2014). Therefore, the stiffness of the culture substrate is a key criterion in biomaterial and culture system selection when optimizing pancreatic differentiation protocols based on insulin-producing cell yield, purity and function (Table 1, Column 6).

One approach could be to mimic the stiffness of the pancreas which may better recapitulate *in vivo* cell function. The stiffness of the adult human pancreas lies within 1.4 ± 2.1–4.4 ± 5.1 kPa (Sugimoto et al., 2014). In contrast, standard tissue culture polystyrene (TCPS) has a stiffness around 3 GPa (Gilbert et al., 2010). Stiffness tunable hydrogels that can be functionalized with ECM proteins, such as polyacrylamide or hyaluronic acid gels, constitute interesting alternatives to polystyrene. Culture of pancreatic progenitors on substrates mimicking the stiffness of the pancreas is a promising strategy to promote differentiation relative to stiff polystyrene. Pancreatic differentiation of hESCs on relatively soft surfaces resulted in increased protein expression of PDX1, and gene expression of pancreatic endoderm markers, *NKX2.2* and *NKX6.1* (Narayanan et al., 2013; Maldonado et al., 2017). Similarly, hESCs encapsulated and differentiated in soft 3D alginate matrices were more viable, proliferative, and had increased PDX1 protein expression suggesting improved pancreatic commitment compared to stiffer gels (Richardson et al., 2016). Significant upregulation of pancreatic associated genes, such as *PDX1*, *INS*, and *GLUT2*, were observed during pancreatic differentiation of hESCs on soft hyaluronic acid hydrogels (Narayanan et al., 2013). hESC-derived beta cells co-cultured with endothelial cells on Matrigel gels formed self-assembled networks of islet organoids with functional glucose-stimulated insulin secretion while these organoids did not form on stiff TCPS coated with Matrigel or in suspension culture (Augsornworawat et al., 2019).

Changing stiffness in hydrogel systems could change the number of cell tethering sites due to increased crosslinking or ECM protein concentration, therefore proper controls are required (Trappmann et al., 2012). To decouple stiffness and substrate binding differences due to composition changes, substrates composed of pillars with controllable aspect ratio can be used to modulate the perceived stiffness while maintaining substrate composition. Nanopillars with a high aspect ratio are more flexible and perceived to be softer compared to those with a low aspect ratio, while maintaining substrate chemistry (Yang et al., 2011). Definitive endoderm differentiation from hESCs commitment is improved on high aspect ratio polycarbonate pillars and resulted in a more cluster-like morphology (Rasmussen et al., 2016b). Controlling cell morphology via substrate stiffness could be another approach to guide differentiation by altering internal cytoskeletal tension, which in turn impacts the compressive forces applied on the cell nucleus thus affecting transcriptional activation (McBeath et al., 2004; Driscoll et al., 2015) (Table 1, Column 7). Transdifferentiation of mouse dermal fibroblasts toward monohormonal pancreatic endocrine cells is improved on soft polyacrylamide gels (1 kPa) and this was correlated with generation of large cell clusters, actin reorganization, and deactivation of YAP compared to culture on stiff substrates (Pennarossa et al., 2018).

During development, the embryo undergoes dynamic changes in ECM stiffness, architecture, and composition. Although optimizing substrates for one stage of differentiation could provide potential insight into developmental mechanics, this may decrease the fraction of pancreatic cells obtained downstream. Using soft, high aspect ratio pillars that promote definitive endoderm differentiation resulted in lower fraction of PDX1^+^ cells compared to flat, polycarbonate controls while the same substrates has decreased downstream pancreatic differentiation (Rasmussen et al., 2016b). Conversely, the formation of PDX1^+^ cells is promoted on soft electrospun fibers while the precursor mesendodermal cell differentiation is improved on stiffer substrates (Maldonado et al., 2017). Alginate encapsulation of single pancreatic progenitor cells at later stages of differentiation promoted an islet-like gene expression profile and increased the fraction of insulin-expressing cells compared to when PSCs were encapsulated at the beginning of differentiation, further suggesting stage specific optimization of these microenvironmental cues is required (Legøy et al., 2020). When comparing pancreatic differentiation results across multiple studies, the classification of “soft” versus “stiff” substrates in relation to the native microenvironment, the type of material, and the ECM used to facilitate cell adhesion must be considered (Table 1, Column 8).

#### ECM Architecture/Topography

The topography of the culture substrate can mediate cell clustering which is thought to promote pancreatic differentiation. The fibrous nature of native ECM can be mimicked using electrospun scaffolds. The ECM provides structural adhesive binding sites which influences the shape cells adopt on a substrate. Scaffolds, such as electrospun nanofibers, can be used to manipulate stem cell fate decisions by mimicking the topography and fibrous nature of native ECM proteins (Xin et al., 2007; Fujita et al., 2012; Kim et al., 2013; Luo et al., 2015). Human ESCs differentiated on 200 nm poly(ε-caprolactone) (PCL) fibers had higher expression of endodermal lineage genes such as *SOX17* and *FOXA2*, compared to on larger fibers. Cells cultured on thin fibers adopted a clumped, rounded morphology while cells on fibers greater than 800 nm were more spread and anchored. Having a more clumped morphology could promote cell–cell interactions over cell–substrate interactions which may bias differentiation (Ghanian et al., 2015). Microporous poly(lactide-co-glycolide) (PLG) and PEG scaffolds can promote aggregation into consistently sized clusters, which improves beta cell differentiation downstream (Youngblood et al., 2019). Culture on nanostructured zirconia substrates promotes clustering of dissociated human pancreatic islets, reduced actin cytoskeletal stress fiber formation, and increased the number of vinculin focal adhesions compared to flat substrates. These traits of zirconia substrates culture were correlated to improved islet survival, growth, and pancreatic differentiation (Galli et al., 2018). These studies suggest that careful selection of culture surface topography could be used to guide tissue organization, which may be a more economical, operator-free alternative to guiding cultures purely with biochemical cues or specialized aggregation techniques.

## Conclusion and Perspectives

Methods to produce functional beta cells from PSCs have advanced by leaps and bounds over the past two decades through advances such as directed differentiation which aimed to mimic the stages of pancreas development. In addition to soluble factors, the interactions between PSC-derived pancreatic cells with each other and with the ECM alter biomechanical stimuli which guide cell fate decision. Therefore, several aspects of the developing microenvironment must be taken into consideration when designing next-generation culture techniques and devices.

For one, understanding the roles of ECM proteins and structure in guiding different stages of differentiation may be crucial in accelerating early stages of differentiation or during transition points in culture. However, these biochemical and structural effects may only have short-term influences on differentiation and may diminish as differentiating pancreatic cells secrete their own native ECM. This speaks to the dynamic nature of the surrounding cellular microenvironment which may temporally vary in both biochemical composition and stiffness. Second, it is important to consider the material and perceived stiffness when interpreting results; a soft substrate in one paper could still be much stiffer than that of the native pancreas. In addition, appropriate design of substrates by controlling stiffness, ECM functionalization, and nanotopography could promote aggregation and pancreatic differentiation. Cell aggregation is an integral part of many differentiation protocols and is clear to be important to obtaining functional beta cells, however, recent studies have shown aggregation may not be required for beta cell differentiation (Hogrebe et al., 2020). Therefore, investigating the functional or phenotypic differences between PSC-derived cells from aggregation culture and 2D protocols, if any, will be important proceeding with scale-up.

Studying and controlling the complex set of factors which guide pancreas development using *in vitro* models is challenging – particularly since stimuli from soluble factors, cell–cell interactions, and cell–ECM interactions are both interrelated and time-dependent. The progression of pancreatic differentiation will likely be dependent on gaining a fundamental understanding of development and then translating this knowledge to scalable technologies. For one, the dynamic nature of maturing cells changing the surrounding microenvironment, and the subsequent effect on differentiation is not well-understood. Complex *in silico* models and improved culture systems may be required to tease apart the many confounded effects of the dynamic cellular microenvironment. Previously, mathematical models of the interactions between biomechanical stimuli and regulatory gene networks have been used to predict MSC (Peng et al., 2017) and pancreatic cell fate decisions (Zhou et al., 2011, 2016; de Back et al., 2013; Wang et al., 2020). Novel *in silico* models which deal with cell generated forces in aggregate culture (Lee et al., 2019) while varying stiffness or cell organization over time could help us understand how mechanotransduction changes throughout a differentiating aggregate. As we move to more complex aggregates and culture systems, *in silico* models of biomechanics could shed light onto correlations between cell differentiation and varying substrate parameters. It is still not well-understood why stem cell-derived beta-like cell co-cultures with endothelial cells (Augsornworawat et al., 2019) have not been able to surpass protocols without co-culture (Nair et al., 2019; Velazco-Cruz et al., 2019; Hogrebe et al., 2020). The presence of flow during culture might be required to recapitulate the effects of endothelial cells on islet development since hemodynamic stimuli have profound impact on endothelial cell function including their secretome (Berthiaume and Frangos, 1995; Burghoff and Schrader, 2011; Wang et al., 2013). In addition, mimicking vascular flow through organized endothelial cell-islet co-cultures could better mimic *in vivo* stimulation, and better reproduce nutrient gradients throughout the aggregates.

**FIGURE 3:**
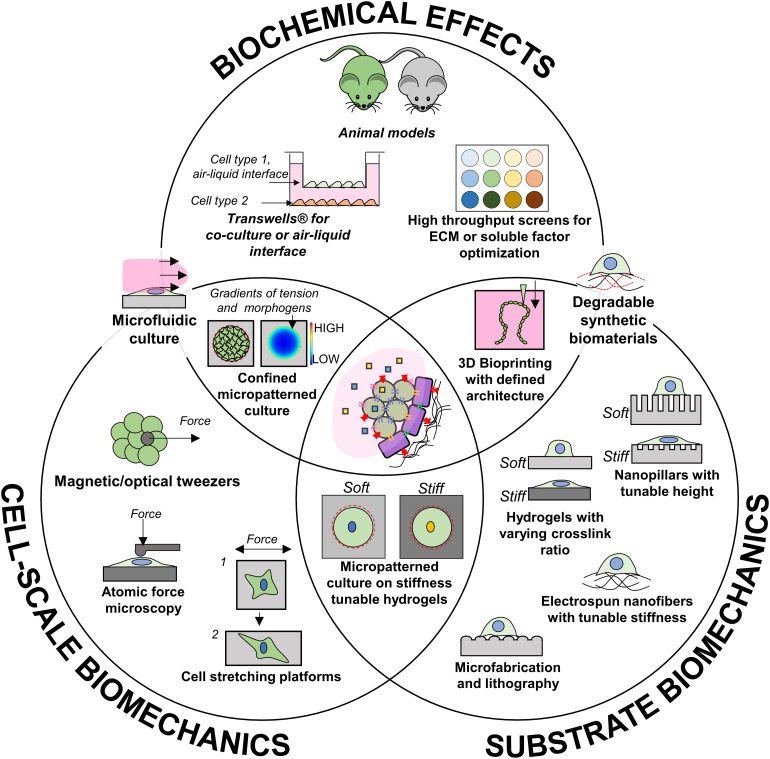
Available technologies that could contribute to investigating independent aspects of pancreas development.

To address these questions, some technologies that could be used to specifically identify the effect of these factors are highlighted in Figure 3 but the state of the art is currently limited in the ability to emulate the temporal changes that occur during development. Once each independent effect is better understood, dynamic culture systems which leverage cell–cell interactions and timed biomechanical cues alongside soluble factor timing could be key for optimal production of functional beta cells. Future *in vitro* models implementing smart materials with dynamically tunable biomechanical properties could give insight to how the changing microenvironment could affect development. For example, synthetic scaffolds with defined ECM functionalization and degradation characteristics would allow for precise control of biochemical, topographical, and stiffness cues presented to differentiating cells. Such a technology could accelerate lengthy differentiation protocols and promote cell morphologies which allow for optimal beta cell organization. Alternatively, novel models which mimic dynamic shape changes in tissue morphogenesis (via novel 4D biomaterials) or stiffness (MMP-mediated degradation or UV-based) could better accommodate the constantly changing requirements for beta cell differentiation. Finally, as our knowledge progresses, increasingly complex co-culture systems involving defined compositions of PSC-derived endothelial, islet endocrine cells, and other cells present during development, may be the route to a functional cell source for islet transplantation.

Overall, better understanding the mechanobiology of pancreas development could lead to new strategies to more efficiently produce functional glucose-responsive, insulin-secreting cells for cell therapies. Interdisciplinary approaches combining advances in materials science and developmental biology could accelerate the development and scale up of culture systems tailored for diabetes cell therapy.

## Author Contributions

RT conducted the literature search and drafted the manuscript in consultation with CM and CH. All authors contributed to writing the final manuscript.

## Conflict of Interest

The authors declare that the research was conducted in the absence of any commercial or financial relationships that could be construed as a potential conflict of interest.
